# Chronicity and Sex Affect Genetic Risk Prediction in Schizophrenia

**DOI:** 10.3389/fpsyt.2020.00313

**Published:** 2020-06-09

**Authors:** Sandra M. Meier, Anna K. Kähler, Sarah E. Bergen, Patrick F. Sullivan, Christina M. Hultman, Manuel Mattheisen

**Affiliations:** ^1^ Department of Psychiatry, Dalhousie University, Halifax, NS, Canada; ^2^ Department of Medical Epidemiology and Biostatistics, Karolinska Institutet, Stockholm, Sweden; ^3^ Stanley Center for Psychiatric Research, Broad Institute of MIT and Harvard, Cambridge, MA, United States; ^4^ Departments of Psychiatry and Genetics, University of North Carolina, Chapel Hill, NC, United States; ^5^ Department of Biomedicine, Aarhus University, Aarhus, Denmark; ^6^ Department of Clinical Neuroscience, Centre for Psychiatric Research, Karolinska Institutet, Stockholm, Sweden; ^7^ Stockholm Health Care Services, Stockholm County Council, Stockholm, Sweden; ^8^ Department of Psychiatry, Psychosomatics and Psychotherapy, University of Wuerzburg, Wuerzburg, Germany

**Keywords:** schizophrenia, polygenic risk score, prediction, sex, course

## Abstract

Schizophrenia (SCZ) is a severe mental disorder with immense personal and societal costs; identifying individuals at risk is therefore of utmost importance. Genomic risk profile scores (GRPS) have been shown to significantly predict cases-control status. Making use of a large-population based sample from Sweden, we replicate a previous finding demonstrating that the GRPS is strongly associated with admission frequency and chronicity of SCZ. Furthermore, we were able to show a substantial gap in prediction accuracy between males and females. In sum, our results indicate that prediction accuracy by GRPS depends on clinical and demographic characteristics.

## Introduction

Schizophrenia (SCZ) is regarded as one of the most serious mental disorders, due to the substantial morbidity, mortality, immense personal and societal costs of the disorder (1–3). Given the serious long-term implications, early detection is of the essence. SCZ has a strong genetic component, and it is nowadays well accepted that a large number of independent loci contribute to the disease, each adding only a small risk (4). A recent advancement in psychiatric genetics has been the use of genomic risk profile scores (GRPS) (5). As a measurement of genetic liability, GRPS aggregate risk alleles from variants identified in genome-wide association studies (GWAS) into a weighted sum that assesses the inherited liability to SCZ. GRPS have been shown to successfully predict case-control status of SCZ, albeit with varying sensitivity and specificity (4). Besides known confounders, such as population stratification, clinical and demographic characteristics of the samples studied might affect prediction accuracy of GRPS. Recently, we showed that GRPS are significantly associated with chronicity of SCZ. In our analyses we demonstrated that oversampling of cases with a high number of admissions significantly improved prediction accuracy (6). Here, we aim to replicate our previous findings from Denmark in a large population-based sample in Sweden that was enriched in more chronic and older SCZ patients in contrast to our original sample (6). Given the differential expression of SCZ in women and men, especially with regard to the clinical course, we also aim to explore potential mediating effects of sex.

## Method

A detailed description of the Swedish cohort with regard to subject ascertainment, diagnosis and validation can be found elsewhere (7). Briefly, ethical committees in Sweden and the US approved all procedures, and all subjects provided written informed consent. Cases with SCZ were identified using the Swedish Hospital Discharge Register (8), which captures all public and private inpatient hospitalizations. Controls were randomly selected from Swedish population registers. In this study, we only included individuals born after 1954 in order to ensure complete coverage for hospital contacts from time of diagnosis to the end of follow-up. The sample included 2,457 cases with SCZ and 2,702 controls from Sweden, and 2,183 of the study subjects were female (897 cases – 1,286 controls) and 2,976 male (1,560 cases – 1,416 controls). At the time of sampling they were on average 50 years old (standard deviation (sd) 7.15) and since the time of diagnosis they were followed for an average of 22 years (sd 8.22). For all cases, we identified the number of inpatient and outpatient contacts, hospitalization length as well as age of onset from the Swedish Hospital Discharge Register (8). On average, cases with SCZ had in total 27 (sd 30.77) contacts with the health care system, 12 (sd 13.72) as inpatients and 19 (sd 25.44) as outpatients with an average hospitalization length of 642 days (sd 932.83; see **Table 1**). GRPS were calculated based on summary statistics (effect allele, effect size) and derived from the 2014 SCZ GWAS from the Psychiatric Genomics Consortium (4), excluding the Swedish sample. More details on genotyping procedures, quality controls, imputation and calculation of GRPS can be found in the SCZ PGC publication (4) as well the original publication that used this data (7). In a case-only design, we assessed the association of GRPS with the number of admissions weighted by follow-up time using a linear regression model adjusting for ancestry principal components (PC), genotyping batch, age at diagnosis and sex. To explore the effects of sex, we stratified the analyses by sex and added an interaction term of GRPS and sex to the linear regression model. In case-control studies, we subsampled cases according to their number of admissions, to explore whether by increasing the chronicity of cases we will observe an improvement in risk prediction comparing these cases to controls. As inclusion criteria, we required above average chronicity (see above): cases had to have at least 27 contacts in total, 12 inpatient contacts, 19 outpatient contacts, or a hospitalization length of at least 640 days. The Nagelkerke pseudo-R < sp > 2 (NkR^2^) was used to compare the amount of variance explained by PC with the percentage of variance explained while adding GRPS. We employed permutation-based tests in order to assess whether oversampling of frequently admitted cases significantly altered (NkR^2^) measurements compared to the overall case-control sample. In each permutation test, we randomly selected 1,000 times a number of cases from the full dataset that matched the number of filtered cases in the subsamples (for more details see (6)). In all of our above-described analyses, we focused on GRPS for p-value threshold of 0.05 as for this threshold the explanatory power was estimated to be most optimal (4).

**Table 1:** Descriptives Chronicity.

	Total		Females		Males	
	M	*SD*	M	*SD*	M	*SD*
Markers of Chronicity						
N_total	27.38	30.77	28.75	33.98	26.6	28.75
N_inpatient	11.51	13.72	11.51	15.71	11.41	12.42
N_outpatient	18.85	25.44	17.23	26.4	15.18	23.09
N_length	641.84	932.83	509.33	743.91	712.69	1016.77

GRPS, genomic risk profile scores; N_total, number of contacts in total; N_inpatient, number of inpatient contacts; N_out, number of outpatient contacts; N_length, hospitalization length; M, mean; SD, standard deviation.

## Results

The hospitalization length was significantly longer in male SCZ patients (males=712.69, females=509.33, B=0.141, P=0.015), whereas female patients had more outpatient contacts (males=15.18, females=17.23, B=-0.155, P=0.002). GRPS for SCZ showed significant associations with the number of admissions for SCZ, in particular for number of contacts in total (B=0.078, P=0.002), inpatient contacts (B=0.073, P=0.001), and hospitalization length (B=0.089, P=0.005). However, there was no significant association observed for GRPS with the number of outpatient contacts (B=0.024, P=0.374), in line with our previous results (6) (see **Table 2**). Stratified by sex, we only observed significant associations of GRPS and number of admissions in males, for number of contacts in total (B=0.079, P=0.008), inpatient contacts (B=0.095, P=0.001), and hospitalization length (B=0.104, P=0.010). In females, no significant associations were observed (number of contacts in total (B=0.082, P=0.074); inpatient contacts (B=0.041, P=0.297); hospitalizations length (B=0.071, P=0.166)). However, we observed no significant interaction of GRPS and sex on number of contacts in total (B=-0.006, P=0.895), inpatient contacts (B=0.036, P=0.381), and hospitalization length (B=0.043, P=0.465; see **Table 3**).

**Table 2:** Association of GRPS With Chronicity.

	PT=0.05		PT=0.1		PT=0.01	
	B	*P*	B	*P*	B	*P*
Markers of Chronicity						
N_total	0.078	0.002	0.071	0.005	0.089	<0.001
N_inpatient	0.073	0.001	0.076	0.006	0.074	0.001
N_outpatient	0.024	0.374	0.018	0.501	0.046	0.077
N_length	0.089	0.005	0.1.03	0.001	0.071	0.022

GRPS, genomic risk profile scores; N_total, number of contacts in total; N_inpatient, number of inpatient contacts; N_out, number of outpatient contacts; N_length, hospitalization length; PT, p-value threshold; B, regression coefficient; P, p-value.

**Table 3:** Association of GRPS With Chronicity by Sex (PT=0.05).

	Females		Males		Interaction	
	B	*P*	B	*P*	B	*P*
Markers of Chronicity						
N_total	0.082	0.074	0.079	0.008	-0.006	0.895
N_inpatient	0.041	0.297	0.095	0.001	0.036	0.381
N_outpatient	0.036	0.448	0.013	0.691	-0.048	0.336
N_length	0.071	0.166	0.104	0.01	0.043	0.465

GRPS, genomic risk profile scores; N_total, number of contacts in total; N_inpatient, number of inpatient contacts; N_out, number of outpatient contacts; N_length, hospitalization length; PT, p-value threshold; B, regression coefficient; P, p-value.

In case-control analyses, enriching the Swedish samples for more frequently admitted cases significantly increased the variance explained by GRPS compared to the full dataset. Enriching cases for number of contacts in total (NkR^2^ = 0.147, P_perm_ = 0.008), inpatient contacts (NkR^2^ = 0.148, P_perm_ = 0.011), and hospitalization length (NkR^2^ = 0.143, P_perm_ = 0.010) significantly improved prediction accuracy. However, no improvement was seen for restricting cases by the number of outpatient contacts (NkR^2^ = 0.127, P_perm_ = 0.235), in line with the results observed in the case-only design. In both males and females, enriching cases for chronicity significantly improved prediction accuracy (data not shown). Interestingly, we observed a strong effect of sex on the overall risk prediction; the variance explained by GRPS was significantly higher in males (NkR^2^ = 0.167) than in females (NkR^2^ = 0.117, P_perm_ <0.001; see **Table 4**).

**Table 4:** Association of GRPS With Subsamples of Cases vs. Controls.

	PT=0.05	
	*NkR* ^2^	*Pperm*
Subsamples		
N_total	0.147	0.008
N_inpatient	0.148	0.011
N_outpatient	0.127	0.235
N_length	0.143	0.010
Females	0.117	0.001
Males	0.167	0.001

GRPS, genomic risk profile scores; N_total, number of contacts in total; N_inpatient, number of inpatient contacts; N_out, number of outpatient contacts; N_length, hospitalization length; PT, p-value threshold; NkR^2^, Nagelkerke pseudo-R^2^; P_perm_, p-value permutation test.

## Discussion

Chronic SCZ has long been hypothesized to index higher familiality and genetic loading. In this study, we report strong associations of GRPS and treatment contacts for SCZ, suggesting an effect of genetic vulnerability on the clinical outcome in SCZ. Specifically, we observed associations with number of contacts in total, inpatient contacts, and hospitalization length. But we did not observe an association with the number of outpatient contacts. In contrast to the other measurements, outpatient contacts are unlikely to configure markers of chronicity, as they are mostly prearranged and reflect with a higher likelihood regular check-ups and not medical needs due to a worsening of symptoms. In line with our findings, familial loading for psychotic disorders was found to deteriorate the course of SCZ (9). This relationship can be more clearly observed in male subjects, although we observed no moderating effect of sex. Interestingly, independent of chronicity the prediction accuracy of GRPS was significantly worse in female subjects with SCZ. While we observed no significant sex-specific effects of GRPS on chronicity, GPRS did not differentiate female cases as well from controls as male cases. This could indicate that female SCZ might be less (or differently) genetically determined and potentially has a stronger contribution of other factors, although GWASs did not find or reveal any sex-specific effects of SCZ so far (7). But the samples included in SCZ GWAS from the Psychiatric Genomics Consortium (4) were also more likely to be male and chronically ill, which might be reflected in our results. Meaning that males and chronic cases are simply better represented in current genetic studies of SCZ and thus more genes might have been identified for these specific subtypes resulting in higher prediction accuracy. In sum, our results demonstrate that prediction accuracy of GRPS can be affected by sample characteristics and thereby inform on potential usage of GRPS in clinical prognosis.

## Ethics Statement

Briefly, ethical committees in Sweden and the US approved all procedures, and all subjects provided written informed consent [see (7)].

## Author Contributions 

SM and MM conceived the idea of the study. MM supervised the analysis pipeline. SM performed the analyses. AK, SB, PS, and CH provided and processed samples. SM and MM wrote the manuscript. All authors discussed the results, and approved the final version of the manuscript.

## Funding

SB was funded through a R21 from the National Institute of Mental Health #MH116188-01. PFS was supported by the Swedish Research Council (Vetenskapsrådet, award D0886501), the Horizon 2020 Program of the European Union (COSYN, RIA grant agreement n° 610307), and US NIMH (U01 MH109528 and R01 MH077139).

## Conflict of Interest

The authors declare that the research was conducted in the absence of any commercial or financial relationships that could be construed as a potential conflict of interest.
